# “Certified … now what?” On the Challenges of Lifelong Learning: Report from an AMEE 2017 Symposium

**DOI:** 10.1080/21614083.2018.1428025

**Published:** 2018-01-25

**Authors:** Carolin Sehlbach, Martin Balzan, Jonathan Bennett, Helena Prior Filipe, Ebbe Thinggaard, Frank Smeenk

**Affiliations:** ^a^ Educational Development and Research, Maastricht University, Maastricht, the Netherlands; ^b^ UEMS President Respiratory Section, Medical Association of Malta, Gzira, Malta; ^c^ University Hospital Leicester, Leicester, UK; ^d^ Hospital of the Armed Forces (HFAR/PL-EMGFA), Hospital SAMS, Lisbon, Portugal; ^e^ Directive Board of the College of Ophthalmology, Portuguese Medical Council (OM), Lisbon, Portugal; ^f^ Copenhagen Academy for Medical Education and Simulation, Copenhagen, Denmark; ^g^ Catharina Hospital, Eindhoven, The Netherlands

**Keywords:** Certification, recertification, continuing professional development, internationalisation

## Abstract

The increasing mobility of patients and healthcare professionals across the countries of Europe has highlighted the wide variations in both medical training, and provision of medical competency and skills. The maintenance of the standards defining competency and skills have national and international implications and have proved challenging for national regulatory bodies. Thus each nation has introduced different types of Continuing Professional Development (CPD), recertification and relicensing systems. At the Symposium entitled: “ ‘Certified … now what?’ On the Challenges of Lifelong Learning” in August 2017 at the Association for Medical Education in Europe (AMEE) annual conference, we reviewed differing European national relicensing systems were reviewed. The review highlighted various lifelong learning and competence assessment approaches using examples from different medical specialties across several European countries.

## AMEE 2017 symposium

During the AMEE Conference 2017, a symposium was held, organised by a group of professionals working within the field of lifelong learning. The aim of the symposium was to present similarities and differences between national recertification systems for medical specialists and to explore mutual challenges, focusing on the question “Certified … now what?” The symposium was open to all AMEE participants, particularly to those who have recently been certified, are enrolled in a recertification process or are committed to lifelong learning. As examples from different medical specialties and different countries were presented, an international and interdisciplinary audience was able to contribute actively.

## Certification of specialists

The departure point for the Symposium was that of certifying the specialist. Here, the differences in training and certification were described. As an example, the length of training was used to illustrate the variation in training and certification that exists across European countries. Ebbe Thinggaard from Denmark compared the time required to achieve certification in obstetrics and gynaecology, which ranged from four years in Bulgaria, Spain or Turkey, to seven years in the UK. Many European countries have a five-year specialty training programme, such as Denmark, Romania and Malta [1]. Although time spent in training may vary, most training programmes are now related to competency-based medical education, and the need for doctors to engage in lifelong learning remains a constant. Both certification practices and initiatives in continuing professional development (CPD) must facilitate future lifelong learning.

The differences in training requirements prompt several questions: Do specialists in each country have the same competencies and skills? What are the effects of working in differing health economies? Having qualified as a specialist, what happens next?

## Recertification of specialists

Various concepts of lifelong learning such as continuing medical education (CME), CPD and recertification were defined in the second presentation of the Symposium. Here, variation also exists in terminology: recertification (Netherlands), revalidation (UK) and Maintenance of Certification (North America). Knowledge deteriorating over time, paired with advances in new knowledge, technologies or treatments explain the primary need for recertification. Secondly, growing public expectations from the medical profession, calls for accountability, and assurance of safe and quality care underpin the urgent societal need for a system of recertification for clinicians [2]. Recertification can serve as a tool to help healthcare professionals to stay up to date and to improve their fitness to practise. Each country, however, operates a different approach; the complete absence of a formal system; voluntary participation; or a mandatory system.

For several countries the standard for recertification involves attaining a minimum number of CME or CPD credits over a specific period of time. Some countries, however, go further and try to assess knowledge, competence and performance by clinical appraisals, site visits or multisource 360-degree feedback in addition to evidence of CME/CPD.

## European examples

In the next part of the Symposium, Frank Smeenk presented the Dutch approach to recertification, followed by Jonathan Bennett who discussed the British revalidation system. Then Martin Balzan gave an overview on CME/CPD in Malta, and Helena Filipe presented the case of Portugal.

### Recertification in the Netherlands

Recertification for all physicians in the Netherlands has been a legal obligation for many years. The requirements for recertification are based on the so-called Law BIG (“Beroepen in de gezondheidszorg”: a law that applies to all workers in health care) and an addendum to this law, “Kaderbesluit” (Decision on framework), in which all additional general requirements for postgraduate medical training, for certification and recertification of all medical specialists are formulated. The requirements in the Kaderbesluit were updated in 2016.

The main aims of these regulations are to safeguard society against clinical malpractice and to improve the quality of practice of all relevant health care workers. The general requirements mentioned in the Law BIG for re-registration for all medical physicians are that, first, all health care workers need to be recertified every five years. Second, in order to be recertified every applicant should be able to demonstrate the following: they should have direct patient contact of at least on average eight hours or more each week. If physicians do not fulfil the requirement, they can compensate for this by education/training.

When physicians fulfil these criteria, they will be recertified. This will be noted in a special “BIG” register which is open to the public. The legislation has defined which registered health care professionals may practise in their respective specialist areas. All registered health care professionals are also subject to a Disciplinary Law. The additional requirements for re-registration for all medical specialists are the following:

1. The working experience (working in their own profession with direct patient contact) in the previous 5 years should be at least on average 16 hours per week or more.
2. Medical specialists must have at least 200 CME and CPD credits during the previous 5 years.
3. Medical specialists should have given their cooperation to and should have submitted themselves and their team/department to a systematic evaluation of the quality of care that is given by this department.
4. They should have taken part in an accredited system in which their functioning is evaluated. This should have resulted in Individual Development Plans (IDP).

Non-adherence may have several consequences depending on its severity:

1. Loss of licence
2. Re-registration for a limited period of time (less than five years) during which period the professional can try to resolve the re-registration problems.
3. Implementation of an Individual Training Programme in an accredited training centre for one to two years. During this period the professional can try to regain his/her registration.

Important bodies for the legal framework in the Netherlands for (re-)registration of all medical physicians are, first, the College Geneeskundige Specialismen (CGS) which sets the rules for (re-)registrations and all medical training centres. These rules must be approved by the Minster of Healthcare, Welfare and Sports. Thereafter they have the status of a law. The second important body is the RGS (Registratiecommissie Geneeskundig Specialisten; General Medical Registry Committee). This body is responsible for enforcement of the rules set by the CGS. The third body in this legal framework is the “Adviescommissie” (Advisory committee for disputes) where one can challenge a decision of the RGS.

### Recertification in the UK

The General Medical Council (GMC), the medical regulation body for the UK, has determined that recertification, or revalidation as it is known in the UK, is based upon the four core domains of Good Medical Practice. These are defined as (1) knowledge, skills and performance; (2) safety and quality; (3) communication, partnership and teamwork; and (4) maintaining trust. Thus the aim is that all facets of a doctor’s practice will be reviewed and assessed by the mandatory revalidation process. The assessment process is based upon an annual appraisal carried out by trained peer appraisers. All aspects of a doctor’s practice must be incorporated into the appraisal process including clinical leadership, research, education and training. Each annual appraisal contributes to a five-year revalidation cycle, which, if completed successfully, results in a relicensing recommendation.

Clinicians are expected to collect a portfolio comprising six types of supporting evidence to be discussed at least once during the five-year appraisal cycle:

1. Continuing professional development
2. Quality improvement activity
3. Significant events
4. Feedback from colleagues
5. Feedback from patients
6. Review of complaints and compliments

The revalidation process requires that each clinician is assigned a “designated body” which is usually the individual’s main employing organisation. The designated body has the responsibility for ensuring that there is an appropriate system for administering appraisal, training appraisers and appointing “Responsible Officers” who assess the outcome of each appraisal. The possible recommendations made by the Responsible Officer to the GMC are (1) approval for relicensing, (2) deferment pending further supporting information, and (3) failure to engage with potential loss of licence, in which case the GMC will inform the clinician that his/her licence is at risk.

### Recertification in Malta

For Malta’s 1000 medical practitioners there is no legal or ethical obligation to perform CME/CPD, based on the assumption that “doctors are capable of looking after themselves”. Since 2001 all doctors working in the public sector (around 750) have some financial incentives, namely a yearly reimbursement of 1100 euros for CME/CPD activities and 2 weeks of annual study leave, normally to attend educational activities locally or abroad. In addition, certified specialists and GPs (around 500) can apply for 2 merit awards of 1100 euros per year. Reimbursement is verified by a specific board. Merit awards encompass a broad spectrum of what are termed “quality improvement initiatives”. All specialists and GPs are clinical and educational supervisors. About half of the specialists/GPs also hold university appointments with an obligation for bedside teaching of medical students.

While all local societies are active in CPD, the Malta College of Family Doctors has introduced a programme of CPD necessary for continued membership of the college, in an effort to encourage doctors in private practice to participate. There are no financial incentives for this category of around 300 GPs apart from tax-deductable expenses. A minority are involved as tutors for students and trainees. The present voluntary CPD is verified mainly by financial accountability. Preliminary results from a local survey indicate that about half of the local medical profession would accept mandatory CME/CPD, while only 27% would oppose. However, there has been scepticism about peer appraisal and examinations.

CPD appears to be alive and healthy in Malta in a voluntary system with financial incentives, although some doctors in private practice may not participate. The mood in Malta appears to be changing with a majority of practitioners favouring a mandatory system, as long as it is restricted to a minimum number of CME points per year, and a minimum number of hours per week.

### Recertification in Portugal

CPD is at the heart of the medical profession’s accountability. There has long been an unwritten contract between society and the medical profession in which commitment to lifelong learning ensures that doctors maintain a safe and high standard of health care [3]. Lifelong learning has been considered as a professional obligation in the code of ethics of the Ordem dos Médicos (OM) – the Portuguese Professional Medical Association [4]. OM represents physicians when policymakers and health authorities formulate health decrees, laws and directives. OM accredits residency programmes and centres, and monitors medical education at all levels except for undergraduates, who are under the tutelage of a university. OM establishes and endorses best practices, promotes a professional code of ethics and is empowered to impose disciplinary measures in cases of malpractice.

There are two central national health authorities under the leadership of the Ministry of Health:

- The General Direction of Health (Direção Geral de Saúde – DGS) [5] which is the public institution that governs and monitors the National Health Plan, implements clinical audits and priority health care programmes, establishes reference centres, recommends standards and guidelines, monitors and reports on patient safety, and regulates blood and blood component issues.
- The Central Administration of Health System (Administração Central do Sistema de Saúde – ACSS) [6], which governs all activities concerning access to health care in Portugal and is the competent authority for the registration and certification (licensing) of health care professionals, comprising doctors, nurses, diagnostic and therapeutic technicians and hospital administrators.

Undergraduate medical education is under the authority of the Ministry of Education, whereas postgraduate medical professionals are the responsibility of the Ministry of Health. A specialist is licensed by ACSS and can only practise if registered by the OM. The specialist can optionally apply for a position of consultant. This requires a minimum period of five years’ practice and qualification after a national examination based on curricular evaluation and proof of competence. The consultant may further qualify as a senior consultant subject to public hospital vacancies.

While undergraduate and postgraduate medical education are clearly structured and regulated, CPD has thus far been based on trust and conducted as a professional obligation according to a code of ethics. Medical education planning and undertaking are voluntary, self-directed and regulated. Specialist practice is regulated by law and doctors are liable to legal challenge and may be sanctioned by the OM. As a non-compulsory credit system, CPD essentially relies on professionals’ reflection and self-assessment. The trend seems to align with the general European model in which the European Union of Medical Specialists (UEMS) [7] acts as a platform for collaborative interaction, programme harmonisation facilitating cooperation, mobility and evidence-based CPD.

## CPD survey

Before the Symposium, an online questionnaire on opinions regarding best practice for CPD and recertification was sent out to more than 300 Maltese, Portuguese and Dutch medical specialists. The data from this survey were presented during the Symposium, engaging the audience in questions such as: What is the aim of recertification – detecting bad apples or striving for excellence? Should CPD and recertification be voluntary or mandatory? What should be assessed, knowledge or skills? These questions will be discussed in a following manuscript.

## Discussion

The panel members of the Symposium discussed the main issues facing CPD/CME delivery and certification in Europe, and highlighted the following points. With increasing need for a culture of accountability and transparency, there is mounting pressure by the general public on legislators, to ensure that all doctors are competent to safeguard patient safety and high-quality care [8]. Therefore, a majority of EU countries have introduced a mandatory CPD system. The European Commission’s report (2015) throws light on the different models of strict mandatory systems like the UK and the Netherlands, mandatory systems with more limited scope such as Portugal, and voluntary systems as in Malta and most of the Scandinavian countries.

In some countries, these systems are controlled by government ministries, and in others they are professionally supervised and regulated. When the profession is seen to fail to deliver adequate regulation, there is concern that government agencies will take over. This may antagonise the medical profession and lead to disruptive loss of professional autonomy with a negative effect on quality of care. Prospective implementation of recertification programmes should reflect professional codes of ethics and experience gleaned from countries and systems that are already established. The question of whether recertification should strive for minimum standards or for continuing improvement of the individual specialist has not yet been answered. The Netherlands, for instance, is currently moving from minimum standards to focusing on the individual’s development, although minimum standards still have to be met. Another key aspect in introducing recertification is balancing individual professional development, job satisfaction and a sense of social accountability. Patients’ needs and opinions should be central to the recertification processes. In addition, all other relevant CPD stakeholders should be engaged in recertification processes.

The financing of recertification processes must also be addressed. A number of national medical associations feel that a mandatory system should be financed by employers and/or state authorities by taxation, and not charged to the individual doctors [9]. Malta, with a voluntary system in place, supports this argument by providing financial support and paid study leave for CPD for doctors in public employment. In the costly mandatory UK system, dedicated time for education is allocated in Government (National Health Service – NHS) contracts. It also has an administrative infrastructure, including software, administrative staff and trained appraisers.

There is no convincing evidence of a causative link between improved health care outcomes and either mandatory or voluntary recertification systems. Data may show improvement in patient outcomes and improved practice, but this may be coincidental. Thus the question remains whether CPD alone or as a component of recertification is most likely to ensure high quality. We are left with national bodies relying upon belief systems of perceived best practice rather than on hard evidence to implement our regulatory processes.
